# PYloroplasty versus No Intervention in GAstric REmnant REconstruction after Oesophagectomy: study protocol for the PYNI-GAREREO phase III randomized controlled trial

**DOI:** 10.1186/s13063-023-07435-5

**Published:** 2023-06-19

**Authors:** Naoya Okada, Yoshihiro Kinoshita, Shoji Nishihara, Takuma Kurotaki, Aya Sato, Kotaro Kimura, Hiroki Kushiya, Kazufumi Umemoto, Shotaro Furukawa, Takumi Yamabuki, Minoru Takada, Kentaro Kato, Yoshiyasu Ambo, Fumitaka Nakamura

**Affiliations:** 1 Department of Surgery and Center of Esophageal Diseases, Teine Keijinkai Hospital, 1-40 Maeda, Teine-Ku, Sapporo, Hokkaido 006-8555 Japan; 2 Department of Surgery, Teine Keijinkai Hospital, 1-40 Maeda1-12, Teine-Ku, Sapporo, Hokkaido 006-8555 Japan

**Keywords:** Pyloroplasty, Esophageal cancer, Esophagogastric cancer, Esophagectomy, Gastric remnant, Gastric reconstruction, Nutrition, Quality of life, Randomized controlled trial

## Abstract

**Background:**

After esophagectomy for esophageal and esophagogastric cancer, more than half of patients have lost > 10% of their body weight at 12 months. In most cases, the gastric remnant is used for reconstruction after esophagectomy. One of the most serious nutritional complications of this technique is delayed gastric emptying caused by gastric remnant mobilization and denervation of the vagus nerve. The aim of the PYloroplasty versus No Intervention in GAstric REmnant REconstruction after Oesophagectomy (PYNI-GAREREO) trial is to analyze the clinical outcome of modified Horsley pyloroplasty (mH-P) as a method of preventing delayed gastric emptying.

**Methods:**

The PYNI-GAREREO trial is designed as an open randomized, single-center superiority trial. Patients will be randomly allocated to undergo gastric remnant reconstruction with mH-P (intervention group) or no intervention (control group) in parallel groups. All patients with esophageal cancer or esophagogastric cancer planning to undergo curative minimally invasive esophagectomy will be considered for inclusion. A total of 140 patients will be included in the study and randomized between the groups in a 1:1 ratio. The primary outcome is the body weight change at 6 months postoperatively, and the secondary outcomes are the nutritional status, postoperative complications, functional outcome, and quality of life until 1 year postoperatively.

**Discussion:**

We hypothesize that mH-P after minimally invasive esophagectomy more effectively maintains patients’ nutritional status than no pyloroplasty.

**Trial registration:**

UMIN Clinical Trials Registry UMIN000045104. Registered on 25 August 2021. https://center6.umin.ac.jp/cgi-open-bin/ctr_e/ctr_view.cgi?recptno=R000051346.

**Supplementary Information:**

The online version contains supplementary material available at 10.1186/s13063-023-07435-5.

## Background

### Background and rationale

Esophagectomy and reconstruction are necessary for the radical treatment of esophageal cancer or esophagogastric junction cancer. Improved outcomes after minimally invasive esophagectomy (MIE) have been observed during the last two decades, and nutritional considerations in these patients have thus become important. Most patients have lost 5 to 12% of their body weight at 6 months postoperatively, and more than half of patients have lost > 10% of their body weight at 12 months [1]. In most cases, the gastric remnant is used for reconstruction; however, delayed gastric emptying (DGE) may be caused by gastric remnant mobilization and denervation of the vagus nerve [2–4].

DGE can induce anastomotic leakage by negative impact pressure from gastric contents. In the long term, low oral intake might worsen patients’ nutritional status and result in decreasing cancer immunity. However, there is no evidence of efficient ways to prevent DGE [5–8]. Therefore, for the prevention of DGE after MIE and gastric remnant reconstruction, we plan to preliminarily perform modified Horsley pyloroplasty (mH-P) [9].

### Objectives

The aim of the PYloroplasty versus No Intervention in GAstric REmnant REconstruction after Oesophagectomy (PYNI-GAREREO) trial is to compare the postoperative change in body weight between patients undergoing mH-P and no intervention (NI). The change in body weight after mH-P mainly reflects the nutritional status caused by the change in oral intake. The primary outcome is the rate of body weight loss at 6 months postoperatively.

## Methods/design

### Trial design

The PYNI-GAREREO trial is designed as an open randomized, single-center superiority phase III trial. Patients will be randomly allocated to undergo gastric remnant reconstruction with mH-P or NI in parallel groups.

## Methods: participants, interventions, and outcomes

### Study setting

The PYNI-GAREREO trial will be conducted in Teine Keijinkai Hospital as a single-center, two-arm, open-label, randomized phase III superiority trial. Table 1 (SPIRIT) shows the schedule of enrollment, interventions, and assessments. The SPIRIT reporting guidelines were used for this study protocol [10]. The SPIRIT checklist is provided as Additional file 1.

**Table 1:** The schedule of enrollment, interventions, and assessments

Time point	Enrollment and allocation	Study period						
		**Post-allocation**						**Close-out**
	*** − 28 days***	**Surgery**	***2 weeks*** ** ± ** ***5 days***	***1 month***	***3 month***	***6 month***	***9 month***	***12 month***
**Enrollment**								
**Eligibility screen**	X							
**Informed consent**	X							
**Allocation**	X							
**Interventions**								
***mH-pyroloplasty***		X						
***No intervention***		X						
**Assessments**								
**Baseline patient characteristics**	X							
**Baseline tumor characteristics**	X							
**Body composition**	X		X	X	X	X	X	X
**Laboratory data**	X		X	X	X	X	X	X
**Postoperative outcome data**		X	X					
**Pathological data**			X					
**Oncological follow-up data**				X	X	X	X	X
**Gastroendoscopy**						X		X
**Computed tomography**				X	X	X	X	X
**QOL questionnaires**			X	X	X	X	X	X

### Eligibility criteria

#### Population

All male or female patients with resectable esophageal carcinoma or esophagogastric junction cancer will be screened for eligibility by two esophageal surgeons. The patients will undergo MIE and gastric remnant reconstruction for radical treatment of esophageal cancer or esophagogastric cancer. Two expert esophageal surgeons (YK and NO) will perform or assist all interventions.

#### TNM clinical classification

The clinical TNM classification will be evaluated by radiological imaging and endoscopy based on the eighth edition of the TNM classification.

The following are the inclusion criteria:

1. Reconstruction by the posterior sternal route
2. No previous laparotomy with a high degree of adhesion (e.g., traumatic incision for trauma surgery, duodenal ulcer perforation, or gastrectomy)
3. Age of ≥ 18 years at the time of providing informed consent
4. Eastern Cooperative Oncology Group performance status of 0 or 1
5. Treatment by thoracoscopic or robot-assisted thoracic MIE
6. Abdominal operation by manual laparoscopy or laparoscopy
7. Anastomosis by the cervical anastomosis technique
8. Full understanding of the study and voluntary provision of written consent to participate in the study

The following are the exclusion criteria:

1. Swallowing problems and poor oral intake associated with swallowing dysfunction
2. Weight loss of ≥ 20% within 6 months before surgery
3. Any synchronous active advanced cancer or diseases that affect the nutritional status
4. Medical history of DGE
5. Simultaneous resection for gastric cancer, other pyloroplasty procedures (e.g., finger bougie technique, Heineke–Mikulicz strictureplasty, or botulinum toxin injection), or Roux-en-Y reconstruction during the trial
6. Clinical condition inappropriate for participation in this study as judged by the patient’s physician

#### Informed consent

Operators and clinical research coordinators (CRCs) will obtain informed consent or assent from potential trial participants. The operators will explain the procedure, and the CRCs will supportively explain the concept of the trial. The operators and CRCs will inform the potential participants of the trial after the operation schedule has been determined; the information will be provided about 2 weeks before the operation date. The operators and CRCs will give the trial document, patient information sheet, and illustrated schema to the participants. At 1 or 2 days after obtaining informed consent from the participants, the CRCs will re-confirm the participants’ understanding and obtain the document of approval.

#### Randomization

After confirmation of the eligibility criteria, registration will be completed by a web-based system to the UMIN Clinical Trials Registry system. The CRCs will enter the registration details into the system. The minimization method will be used for randomization. The staff member who will assess the blinded medical reports will also be blinded. Patients will be randomized to the intervention group or control group in a 1:1 ratio using a computerized randomization tool (UMIN INDICE cloud) with the minimization method, balancing the arms according to age, clinical TNM stage, and change in preoperative body weight during the most recent 6 months before surgery. The two arms will be the mH-P arm (intervention) and the NI arm (control). Because this trial will involve different surgical techniques, complete blinding of the treating surgeons and medical staff is not feasible. The surgeon will know which operation has been performed, and the medical staff can determine whether the patients have undergone pyloroplasty or not by reading the official medical records. However, a study team member will assess blinded medical reports regarding mH-P or NI to ensure a blinded assessment of the primary outcome parameter.

#### Pyloroplasty procedure

For gastric remnant reconstruction, mH-P either will or will not be performed according to the randomization result. mH-P will be performed by first longitudinally cutting all gastric wall layers to a length of 3 cm at 1.5 cm above and below the pyloric ring; the surgical wound will then be closed using seven to nine absorbable monofilament sutures (4–0 PDS®) in a horizontal Gambee pattern. Intraoperative botulinum injection and application of a pyloric ring finger bougie will be prohibited during the trial.

#### Decompression tube placement and jejunostomy

We will routinely perform decompression tube placement and jejunostomy of the gastric remnant. After mobilization of the stomach, we will cut the gastric serosa and muscular layer. We will then cut the point of the mucosa layer and insert two tubes: the decompression tube (inserted cranially) and the enteral tube (inserted caudally toward the jejunum). The two tubes will be placed in the gastric remnant wall by the Witzel procedure. Finally, after the reconstruction and cervical anastomosis, we will perform abdominal wall plasty of the gastric remnant with the placement of tubes.

#### Criteria for discontinuing or modifying allocated interventions

If non-inferiority of the mH-P group to the NI group for the primary endpoint is demonstrated, and even if superiority is demonstrated in the interim, the study will be stopped (active discontinuation). In such a case, the need for discontinuation of the trial will be comprehensively considered without being restricted by the statistical judgment of the trial or other factors. If the trial is discontinued, the results will be reported. The enrolled patients will be followed up for 1 year.

#### Provisions for post-trial care

In the event that a research participant suffers health damage as a result of the conduct of the research, the physician in charge of the research will provide appropriate treatment and investigate the cause of the damage. In such cases, if treatment or examination becomes necessary, it will be conducted within the scope of the research participant’s normal insurance treatment. Examples of health damage include dumping syndrome or bile reflux after pyloroplasty as well as leakage at the pyloroplasty point.

### Outcomes

The primary endpoint is the rate of body weight loss at 6 months after surgery. The secondary endpoints are oral intake and change in nutritional status, which will be checked during the perioperative hospitalization period and at 1, 3, 6, 9, and 12 months after surgery.

Oral intake will be calculated as a percentage of total energy expenditure, which will be calculated based on the weight and activity coefficient of each patient, and data will be collected at each time point. As the nutritional status of the patient will be monitored by body composition which is analyzed by a bioimpedance analyzer (InBody S10®). The analyzer determines the total body fat mass (kg) and skeletal muscle index (kg/m^2^). Additionally, blood tests will be performed at fixed points to measure nutritional evaluation indices (albumin, pre-albumin, cholesterol, total protein, lymphocyte count, and C-reactive protein).

Intraoperative factors such as the total blood loss, operation time, thoracic approach, subtotal gastric remnant or gastric tube reconstruction, and inclusion or exclusion of Kocher mobilization will also be analyzed.

To evaluate the degree of pyloric transit as a secondary endpoint, the amount and nature of decompressed gastric drainage and the pH and bilirubin level of the drainage will be measured on postoperative days 1, 3, 5, and 7. On day 7, the degree of pyloric drainage and the presence or absence of reflux will be assessed by oral contrast according to the standard clinical path. The blood and gastric drainage measurements will be taken in the laboratory, the data will be recorded in the medical record, and the sample will be discarded. The decompression tube will be clamped at 6:00 am to equalize the condition of the reconstructed elevated stomach, and contrast examination will be performed at 9:00 am on the same day. The contrast medium will be barium diluted to 60%.

The pyloric transit (an objective index) as evaluated by oral barium contrast will be scored according to the presence or absence of gastric contents in the reconstructed elevated stomach at the beginning of the examination (0: no, 1: yes), the pyloric transit time of the contrast medium (0: < 1 s, 1: > 1 s), and stagnation of the contrast medium in the stomach (0: completely disappears; 1: more than half remains; 2: all remains).

During this pyloric transit evaluation, whether the reconstructed gastric remnant has shifted to the thoracic cavity side will also be evaluated by the contrast examination. This examination may result in the performance of intraoperative thoracotomy via the mediastinum during the retrosternal route-creating procedure.

At 1, 3, 6, 9, and 12 months postoperatively, the above parameters and the amount of residue in the reconstructed raised stomach will be evaluated by computed tomography. At 6 and 12 months postoperatively, the amount of residue in the reconstructed raised stomach and the pyloric transit will be evaluated by endoscopy.

Postoperative gastrointestinal symptom questionnaires will be requested at the time of discharge, at the first outpatient visit after discharge and at 3, 6, 9, and 12 months after surgery (Table 2) [11, 12]. Quality of life will be assessed using the evaluated 26 questionnaires (Table 2) by an investigator who is not part of the surgery team. The questionnaires contain 26 items to test eight elements of quality of life (physical performance, reduction in daily activity through physical or mental problems, social activity, mental health, vitality, pain, and global health status). Items are rated on a 5-point scale (1 = never to 5 = always).

**Table 2:** PYNI-GAREREO study questionnaire form

Questionnaire items	Scores				
	(1) Never	(2) Infrequently	(3) Sometimes	(4) Often	(5) Always
Do you have problems eating solid foods (rice, side dishes, etc.) or hard foods?	□	□	□	□	□
Do you have problems eating soft foods such as liquid foods (porridge, etc.)?	□	□	□	□	□
Do you have problems taking a drink?	□	□	□	□	□
Do you have difficulty swallowing saliva?	□	□	□	□	□
Do you choke when swallowing?	□	□	□	□	□
Do you ever have trouble enjoying meals as much as you did before the surgery?	□	□	□	□	□
Do you feel immediately full?	□	□	□	□	□
Do you have trouble eating in front of others?	□	□	□	□	□
Do you have a dry mouth?	□	□	□	□	□
Do you find that food or drinks taste different than usual?	□	□	□	□	□
Do you have trouble coughing?	□	□	□	□	□
Do you ever have trouble having a conversation?	□	□	□	□	□
Do you have trouble burping?	□	□	□	□	□
Do you have trouble with acidity or bitterness rising to your mouth?	□	□	□	□	□
Do you feel pain anywhere when eating?	□	□	□	□	□
Do you experience chest pain when eating?	□	□	□	□	□
Do you experience pain in the area around the solar plexus when eating?	□	□	□	□	□
Do you break out in a cold sweat within about 30 min after eating?	□	□	□	□	□
Do you experience palpitations within approximately 30 min after eating?	□	□	□	□	□
Do you experience dizziness within approximately 30 min after eating?	□	□	□	□	□
Does your stomach rumble within about 30 min after eating?	□	□	□	□	□
Do you have abdominal pain within about 30 min after eating?	□	□	□	□	□
Does your whole body become sluggish and weak 2 to 3 h after eating?	□	□	□	□	□
Do you feel sleepy 2 to 3 h after eating?	□	□	□	□	□
Do you break out in a cold sweat 2 to 3 h after eating?	□	□	□	□	□
Do you have diarrhea?	□	□	□	□	□

The short-term outcomes evaluated in this study will be postoperative complications and the length of hospital stay. Postoperative complications in this trial are defined as anastomotic leakage, stricture, pneumonia, recurrent nerve palsy, surgical site infection, and chylothorax.

The routinely placed jejunostomy enteral tube will be removed within 3 months postoperatively in the outpatient clinic when the patient has been determined to have no risk of reduced oral intake. If the tube feeding is used for a longer period, the prolonged time will be one of the outcome parameters.

Long-term recurrence and the prognosis will also be followed up.

Patients will be blinded to which group they are in, but the physician who performs the surgery and performs the imaging and follow-up will not be blinded to whether pyloroplasty is performed. The dietitian, nurse, and data analyst who evaluate the nutritional status and gastrointestinal symptoms will be blinded to which group each patient is in.

### Sample size

The primary endpoint of this trial is the determination of the rate of change in the postoperative body weight. The sample size required to predict the number of patients necessary for statistical validity (two-sided test) is based on our retrospective data from June 2019 to June 2020 (*n* = 20). These are unpublished data. According to these data, the average rate of body weight loss at 6 months postoperatively is − 4.2% in the mH-P group and − 8.2% in the NI group. The incidence rate of the average body weight loss in the mH-P group is expected to be ≤ 4%. We calculated that 60 patients will be required in each arm of this study with a significance (*α* level) of 0.05 and power (1 − *β*) of 0.8. Anticipating a 5% rate of loss to follow-up, we calculated that 70 patients will be required in each arm of this study (total study population of 140 patients).

### Recruitment

Surgeons and CRCs will check the inclusion and exclusion criteria 1 to 2 days before an eligible patient visits their outpatient clinic. Inclusion rate feedback will be provided every 6 months in a monitoring report. Completeness of case record form (CRF) data and adherence to the study protocol will be checked on a weekly basis by the coordinating investigator or CRC. This trial is advertised to make potential participants aware of and thus recruited by the study outline published on the Teine Keijinkai Hospital homepage.

### Data collection and management

A participant number will be generated upon each patient’s inclusion in the study, and this number will be used for further identification in the database. The participant number key is accessible by the coordinating investigator. Clinical data will be collected by the study coordinator or clinical research nurse and will be recorded in a good clinical practice-compliant digital CRF and database. All non-electronic items containing data will be kept in locked cabinets at the data coordinating centers.

The data will be able to be accessed by the research nurse and research physician. After the study has been completed, requests to access the dataset can be submitted to the project leader or principal and coordinating investigator. The completed CRFs will also be checked with the source data regarding the primary outcome parameter and important secondary outcome parameters.

Data will be collected via datasheets on paper and kept securely. All handling cases will be managed by subject identification code or anonymized registration number. The correspondence table of the anonymizing code and names and the consent form containing the names will be kept strictly in the separate lockable document storage at Teine Keijinkai Hospital Clinical Research Center.

### Follow-up

For each participant, the study will start at randomization, and the patient will be followed until 12 months after surgery. The primary outcome parameter will be evaluated every 3 months after surgery. During the 1 year of follow-up, data on readmission, functional results, and quality of life will be generated. Study visits will be scheduled to take place 4 weeks before surgery and 1, 3, 6, 9, and 12 months after surgery. The functional outcomes are the pyloric transit, bile reflux, oral intake, and gastrointestinal symptom questionnaires.

### Statistical methods

The Mann–Whitney *U*-test will be used to compare continuous variables such as weight change, which is the primary endpoint; body composition; and blood test indices. Student’s *t*-test (two-tailed) will be used to compare the mean parameters, and the chi-square test will be used to compare the bivariate variables such as the presence of perioperative complications.

Further subgroup analysis will only be carried out in case of significant interaction effects. The Wilcoxon signed rank test will be used to determine significant effects. Comparisons between the mH-P and control group for weight change either pre- or postoperative 6 months will be performed. Differences with a *p* < 0.050 are considered statistically significant.

### Interim analyses

Two interim analyses will be performed during the course of the study to determine whether the primary objective of the study has been achieved. The first interim analysis will be performed during enrollment to determine whether it is appropriate to continue enrollment, and the second interim analysis will be performed early after the end of enrollment to determine whether to continue follow-up for the planned period. In either case, if it is determined that the primary objective of the study has been achieved, the study will be terminated, and the results will be promptly published in conferences and papers.

The first interim analysis will be conducted using data from periodic monitoring when half of the expected number of enrolled patients have completed 1 year of follow-up. The second interim analysis will be conducted in conjunction with periodic monitoring at a time deemed appropriate after consultation between the data center and the CRC, around the time when enrollment is completed, and protocol treatment is completed for all enrolled patients. In principle, enrollment will not be stopped during the first interim analysis. The decision criteria based on the results of the interim analysis of this study will be as follows. If the non-inferiority of the mH-P group to the NI group for the primary endpoint is not demonstrated, or if non-inferiority is demonstrated but superiority is not demonstrated, the study will be continued in either case. If non-inferiority of the primary endpoint of mH-P over NI is demonstrated and superiority is also demonstrated, the study will be terminated (effective termination). If the primary endpoint of the mH-P group is lower than that of the NI group, the need for discontinuation of the study will be comprehensively considered without being restricted by statistical judgment such as tests (invalid discontinuation).

### Oversight and monitoring

An independent data and safety monitoring committee will evaluate the progress of the trial and examine safety variables. The committee will consist of a surgeon, CRCs, and a statistician. Individualized patient description charts including safety parameters will be presented to the committee, including one table comprising these endpoints in blinded groups every 6 months. The main safety parameters are all serious adverse events (AE): mortality, multiple organ failure, anastomotic leakage, pulmonary complications, cardiovascular complications, reinterventions, and reoperation. After the investigators have presented the data, the members of the committee will discuss these results in the absence of the investigators and will then advise them. Possible options will include continuing the trial, performing an interim analysis, adjusting the trial’s design, and discontinuing the trial. Discontinuation will be advised if the committee concludes that the results would convince a broad range of clinicians that one trial arm is inferior or if safety is compromised in one arm. If the committee advises adjustment of the trial’s design, performance of an interim analysis, or discontinuation of the trial, the responsible medical ethical committee will also be notified. Serious adverse events (SAE) will be reported to the chairman of the hospital. The PYNI-GAREREO trial will be monitored according to the Japanese ethical guidelines for medical and health research involving human subjects. This is a low-risk study because both interventions being investigated are considered to be standard care in Japan.

In-house monitoring will be performed every year by a third party to evaluate and improve the progress and quality of the study. A Patient and Public Involvement (PPI) member will not be part of the committee.

The first interim analysis will be conducted after half of the planned number of patients are enrolled, and the second interim analysis will be conducted immediately before the planned patient accrual is completed. The data and safety monitoring committee will review the interim analysis reports independently from the investigators and statistician. In-house monitoring will be performed every 6 months by a data center to evaluate and improve the study progress, data integrity, and patient safety.

### Safety

AE are defined as harmful events that occur in the study population during the research period, without any intervention. Any untoward medical occurrence in a patient or surgical intervention as clinical investigation does not necessarily have to have a causal relationship with this treatment. An AE can therefore be any unfavorable and unintended sign (including an abnormal laboratory finding, for example), symptom, or disease temporally associated with the intervention, whether considered related to the medicinal or investigational. SAE is defined as any untoward medical occurrence that meets any of the following criteria: harmful events result in death, are life-threatening, result in persistent or significant disability/incapacity, require inpatient hospitalization or prolongation of existing hospitalization, and are congenital anomaly/birth defects. Examples of harmful events in this study include mortality, multiple organ failure, major anastomotic leakage, pulmonary and cardiovascular complications, reinterventions, and reoperations.

An important medical event that may not result in death, be life-threatening, or require hospitalization may be considered an SAE when, based upon appropriate medical judgment, the event may jeopardize the subject’s health and may require medical or surgical intervention to prevent one of the outcomes listed in this definition. SAE timeline is 24 h. AE/SAE assessment will start from the first treatment. CRC and monitoring doctors will assess the AE/SAE severity, causality, and expectedness. AE/SAE will be reported on paper CRF. AE/SAE data will be collected in this study, but SAE will be reported to the hospital chairman and ethical committee. Patient safety data will be checked every 6 months through in-house monitoring of hospital and outpatient courses, including patients’ symptoms, unplanned laboratory tests, and modality tests.

A serious breach is defined as a breach of the law or regulations, or the protocol or other document referred to in them, which is likely to affect to a significant degree, the safety or physical or mental integrity of the trial subjects, the scientific value of the trial, or the quality or integrity of the data generated in the trial. In case of serious breaches, we will report to CRC, the chairman of the Teine Keijinkai Hospital, and the Ministry of Health, Labour and Welfare of Japan within 2 days.

### PPI

**Fig. 1:**
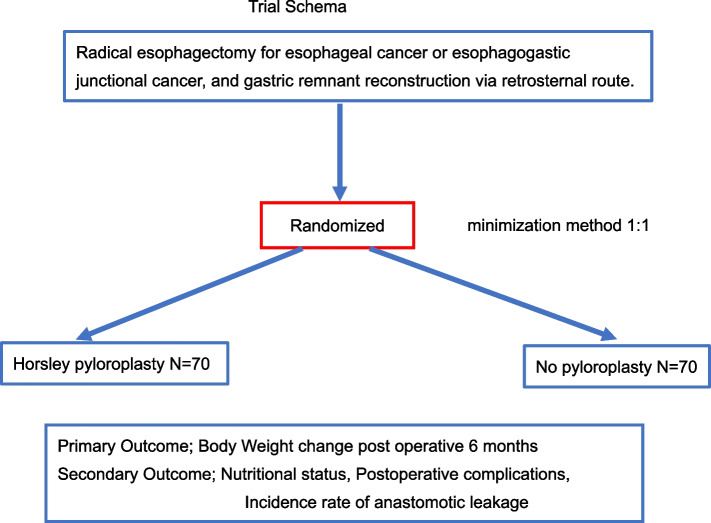
Trial schema

PPI will contribute to the study design and the review of informed consent forms. The study will be advertised on the Teine Keijinkai Hospital homepage to recruit potential participants, and any questions they have will be answered by the CRCs and doctors (Fig. 1, Table 3).

**Table 3:** Trial registration data

Data category	Information
Primary registry and trial identifying number	UMIN Clinical Trials Registry R000051346, UMIN000045104 (https://center6.umin.ac.jp/cgi-open-bin/ctr_e/ctr_view.cgi?recptno=R000051346)
Date of registration in primary registry	2 September 2021
Secondary identifying numbers	Teine Keijinkai Medical Center Medical Ethics Committee Approval 2–021029-00
Source(s) of monetary or material support	Teine Keijinkai Hospital
Primary sponsor	Teine Keijinkai Hospital
Contact for public queries	NO, MD, PhD okada-na@keijinkai.or.jp
Contact for scientific queries	NO, MD, PhD okada-na@keijinkai.or.jp Department of Surgery & Center of Esophageal Diseases, Teine Keijinkai Hospital, Sapporo, Japan
Public title	Pyloroplasty versus no intervention in gastric remnant reconstruction after esophagectomy
Scientific title	Pyloroplasty versus No Intervention in GAstric REmnant REconstruction after Oesophagectomy: study protocol of the PYNI-GAREREO randomized controlled trial
Countries of recruitment	Japan
Health condition(s) or problem(s) studied	Delayed gastric emptying, nutritional complications, gastric remnant reconstruction after esophagectomy
Intervention	Modified Horsley pyloroplasty
Key inclusion and exclusion criteria	Inclusion criteria: (1) Reconstruction by the posterior sternal route (2) No previous laparotomy with a high degree of adhesion (e.g., traumatic incision for trauma surgery, duodenal ulcer perforation, or gastrectomy) (3) Age of ≥ 18 years at the time of providing informed consent (4) Eastern Cooperative Oncology Group performance status of 0 or 1 (5) Treatment by thoracoscopic or robot-assisted thoracic MIE (6) Abdominal operation by manual laparoscopy or laparoscopy (7) Anastomosis by the cervical anastomosis technique (8) Full understanding of the study and voluntary provision of written consent to participate in the study
	Exclusion criteria (1) Swallowing problems and poor oral intake associated with swallowing dysfunction (2) Weight loss of ≥ 20% within 6 months before surgery (3) Any synchronous active advanced cancer or diseases that affect the nutritional status (4) Medical history of DGE (5) Simultaneous resection for gastric cancer, other pyloroplasty procedures (e.g., finger bougie technique, Heineke–Mikulicz strictureplasty, or botulinum toxin injection), or Roux-en-Y reconstruction during the trial (6) Clinical condition inappropriate for participation in this study as judged by patient’s physician
Study type	Interventional
	Open randomized, single-center superiority trial. Patients will be randomly allocated to undergo gastric remnant reconstruction with modified Horsley pyloroplasty or no intervention in parallel groups
	Primary purpose; prevention
	Phase III
Date of first enrollment	2 September 2021
Target sample size	140; 70 vs 70
Recruitment status	Recruiting
Primary outcomes	Body weight change at 6 months postoperatively
Key secondary outcomes	Nutritional status, postoperative complications, functional outcome, and quality of life until 1 year postoperatively

## Discussion

MIE is necessary for the radical treatment of esophageal cancer and esophagogastric junction cancer. The most common reconstruction organ after MIE is the gastric remnant [13]. Approximately 10 to 15% body weight loss 1 year after esophagectomy has been reported [1, 14]. There are manifold reasons for aberrant nutrition after esophagectomy, including altered anatomy; early satiety; loss of appetite, taste, and smell; and postsurgical dumping syndrome [11]. DGE and pyloric stenosis, which are causes of changes in the nutritional status, are potentially caused by gastric remnant mobilization and denervation of the vagus nerve [15]. DGE might also induce anastomotic leakage by negative impact pressure from gastric contents [16]. In the long term, low oral intake caused by DGE might worsen the nutritional status and result in decreasing cancer immunity [1, 17].

One method of preventing DGE is pyloroplasty. Although some reports and systematic reviews have shown the clinical efficacy of pyloroplasty, various pyloroplasty procedures were evaluated (e.g., finger bougie technique, Heineke–Mikulicz strictureplasty, and botulinum toxin injection) [6, 8, 18–21]. There is no definitive evidence of efficient ways to prevent DGE, especially when performing gastric remnant reconstruction via the retrosternal route [22]. To prevent pyloric stenosis and/or DGE after esophagectomy, we plan to preliminarily perform mH-P [9]. The purpose of this study is to determine whether mH-P is clinically effective in terms of postoperative oral intake and nutritional status. We will investigate the improvement of pyloric passage and incidence of anastomotic leakage after esophagectomy and reconstruction, limiting the procedure to gastric remnant reconstruction via the retrosternal route, as well as the effect of maintaining the oral intake and nutritional status in a randomized controlled trial of mH-P versus NI.

In Japan, the national clinical data regarding short-term outcomes after surgery for esophageal cancer show that the postoperative surgery-related mortality rate ranges from 1.6 to 2.8% [23], and the future focus on care has shifted to survival with high quality of life. Nutritional considerations in these patients represent one of the greatest contributors to quality of life. If this study proves that mH-P can contribute to the improvement and maintenance of the nutritional status, this pyloroplasty method might become the standard additional procedure.

## Trial status

The trial has been recruiting since September 2021. This trial recruitment will end when the planned number of patients have been enrolled or depending on the results of the interim analyses. We plan to conduct two interim analyses. The first interim analysis will be conducted after half of the planned number of patients have been enrolled, and the second interim analysis will be performed immediately before the planned patient accrual has been completed. The data and safety monitoring center will review the interim analysis reports independently from the group investigators and group statistician. If the superiority of the mH-P group is demonstrated with an adjusted α level, the study will be terminated. In-house monitoring will be performed every 6 months by the data center to evaluate and improve the study progress, data integrity, and patient safety.

## Supplementary Information

**Additional file 1. **SPIRIT Checklist. Completed SPIRIT checklist for the current study protocol. Funding declaration. Ethical approval document. Approval by the medical ethics committee. Consent form for clinical trial.

## Data Availability

The clinical statistician and clinical research coordinators have access to the final trial dataset. The investigators are limited to such access. The datasets analyzed during the current study, the statistical code, and the full protocol are available from the corresponding author upon reasonable request.

## Abbreviations

MIE: Minimally invasive esophagectomy
DGE: Delayed gastric emptying
mH-P: Modified Horsley pyloroplasty
NI: No intervention
CRC: Clinical research coordinator
CRF: Case record form
AE: Adverse event
SAE: Serious adverse event
PPI: Patient and Public Involvement
